# Comparative effects of non-specific exercise therapies on adolescent idiopathic scoliosis: a systematic review and network meta-analysis

**DOI:** 10.3389/fspor.2026.1901060

**Published:** 2026-09-25

**Authors:** Yaxin Guan, Zening Zhang, Shu Xie

**Affiliations:** School of Exercise and Health, Shanghai University of Sport, Shanghai, China

**Keywords:** adolescent idiopathic scoliosis, Cobb angle, core muscle group training, network meta-analysis, non-specific exercise therapy

## Abstract

**Objective:**

Non-specific exercise therapies are widely used in the conservative management of adolescent idiopathic scoliosis (AIS) because of their accessibility and broad applicability. However, the comparative effectiveness of different non-specific exercise modalities remains unclear. This study aimed to systematically review the available evidence and compare the effects of different non-specific exercise therapies on AIS using network meta-analysis.

**Methods:**

PubMed, Embase, Web of Science, the Cochrane Library, CNKI, Wanfang, and VIP databases were searched from inception to September 2025. Eligible studies included participants diagnosed with AIS aged 10–19 years. Non-specific exercise therapy was defined as an exercise-based intervention that was not individualized according to curve type or formal three-dimensional scoliosis classification and did not involve scoliosis-specific correction principles. The primary outcome was Cobb angle. Pairwise meta-analysis was conducted using RevMan 5.4, and network meta-analysis was conducted using Stata 18.0. Treatment rankings were estimated using SUCRA values.

**Results:**

The overall pairwise random-effects meta-analysis suggested that non-specific exercise therapies were associated with a greater reduction in Cobb angle than control conditions (MD = −5.28, 95% CI −9.25 to −1.30). However, substantial heterogeneity was observed across studies (I^2^ = 98%), and the 95% prediction interval was wide and crossed the null value (95% PI: −20.18 to 9.62). Therefore, this pooled estimate was interpreted as an exploratory summary of the overall direction of direct evidence rather than as a definitive treatment effect. In the primary network meta-analysis, spinal flexibility training and core muscle group training showed higher ranking probabilities according to SUCRA values. Nevertheless, these rankings should be interpreted cautiously because the evidence network was sparse, several comparisons were informed by a limited number of studies, and concerns remained regarding heterogeneity, risk of bias, transitivity, and confidence in network evidence.

**Conclusion:**

Non-specific exercise therapies may be associated with reductions in Cobb angle in adolescents with idiopathic scoliosis. Spinal flexibility training and core muscle group training appeared to rank higher in the primary network analysis, but the certainty of these findings is limited by substantial heterogeneity, small sample sizes, sparse network structure, methodological limitations, concerns regarding transitivity, and the inclusion of non-randomized studies.

**Systematic Review Registration:**

https://www.crd.york.ac.uk/PROSPERO/view/CRD420251242017, PROSPERO: CRD420251242017.

## 1 Introduction

Adolescent idiopathic scoliosis (AIS) is a three-dimensional spinal deformity that develops during puberty, featuring a lateral curvature of the spine accompanied by vertebral rotation. It is diagnosed after excluding congenital, neuromuscular, and other secondary causes (1). AIS affects approximately 1.5%–3% of adolescents and accounts for over 80% of all scoliosis cases (2), representing a major musculoskeletal concern during growth.

Recent epidemiological surveys indicate that the prevalence of AIS is increasing gradually worldwide and exhibits clear sex and region-related differences. Population-based school screening programs consistently report higher detection rates in girls than in boys, with prevalence ranging from 2% to 9% depending on age and screening criteria (3–5). These findings highlight the growing public-health burden of AIS and underscore the need for effective conservative management strategies.

Without appropriate intervention, AIS may lead to impaired postural development, reduced pulmonary function, decreased physical performance, and psychosocial distress (6). Surgery is generally reserved for curves exceeding 40°, offering structural correction but carrying higher risk and longer recovery (7). For patients with mild-to-moderate AIS, non-surgical approaches remain the first-line treatment, among which exercise-based interventions are widely used due to their safety, accessibility, and potential to improve posture and motor control.

Exercise-based interventions for AIS are commonly divided into specific and general (non-specific) programs. Specific approaches, most notably the Schroth method, target curve-specific postural corrections and have well-documented benefits (8, 9). However, they require trained therapists and specialized facilities, which may limit accessibility. In contrast, general exercise programs—such as core stabilization, flexibility training, proprioceptive exercises, or corrective gymnastics—are not curve-type dependent and can be implemented in schools, community settings, or at home. These features make general exercise interventions particularly suitable for adolescents with mild curves or for settings with limited medical resources (10).

Although numerous studies have investigated the effects of individual exercise modalities, evidence regarding the comparative effectiveness of different general exercise programs for AIS remains fragmented. Existing systematic reviews primarily focus on specific exercise approaches, and few provide a quantitative comparison across multiple non-specific interventions. Therefore, a comprehensive synthesis and comparative evaluation, ideally through a network meta-analysis, is needed to clarify their relative benefits and guide clinical decision-making (11).

To address this evidence gap, we conducted a systematic review and network meta-analysis to compare the effects of different non-specific exercise-based interventions for AIS. The primary outcome was Cobb angle, which is the most widely used radiographic indicator of curve severity. By synthesizing direct and indirect evidence, this study aimed to summarize the current evidence landscape, explore the relative effects of different non-specific exercise modalities, and identify areas where future high-quality trials are needed.

## 2 Research methodology

This review was prospectively registered in PROSPERO (CRD420251242017) and conducted in accordance with the PRISMA 2020 guidelines (12). Literature searching, study selection, and data analysis were completed on September 14, 2025.

### 2.1 Search strategy

A comprehensive search was performed in PubMed, Embase, Web of Science, the Cochrane Library, CNKI, Wanfang, and VIP databases from inception to September 2025. The search strategy combined Medical Subject Headings (MeSH) and free-text terms related to AIS (e.g., “adolescent idiopathic scoliosis,” “juvenile idiopathic scoliosis,” “scoliosis”) and exercise-based interventions (e.g., “exercise therapy,” “core stability,” “flexibility training,” “proprioceptive training,” “yoga,” “Pilates,” “aerobic exercise,” “corrective exercise”). Boolean operators (“AND,” “OR”) were used to construct the search strings. No language restrictions were applied during the search. However, only English- and Chinese-language studies met the final eligibility criteria. The complete search strategies for each database are provided in Supplementary Table S1.

### 2.2 Inclusion and exclusion criteria for literature selection

#### 2.2.1 Inclusion criteria

Studies were included if they met all of the following criteria:

1. participants were diagnosed with adolescent idiopathic scoliosis (AIS) and were aged 10–19 years;
2. the intervention group received a non-specific exercise therapy. In this review, non-specific exercise therapy was operationally defined as an exercise-based intervention that was not individualized according to curve type or three-dimensional scoliosis classification and did not require formal scoliosis-specific correction principles, such as Schroth-based rotational angular breathing or curve-specific auto-correction. Eligible non-specific exercise interventions included general core stability training, spinal flexibility training, Pilates- or yoga-based general flexibility exercises, proprioceptive or motor-control training, aerobic or resistance training, and school- or home-based general corrective gymnastics;
3. the control group received routine care, no intervention, regular physical education, or conventional non-specific exercise; and
4. the study reported at least one relevant outcome, including Cobb angle, angle of trunk rotation, scoliosis severity, pulmonary function, quality of life, or physical function.

#### 2.2.2 Exclusion criteria

Studies were excluded if they met any of the following criteria:

1. reviews, conference abstracts, case reports, single-case studies, or other non-original studies;
2. studies without accessible full texts;
3. studies with incomplete or missing data that precluded qualitative or quantitative synthesis;
4. studies involving postoperative rehabilitation;
5. studies including participants outside the predefined age range of 10–19 years, unless data for the eligible age subgroup could be extracted separately;
6. studies evaluating scoliosis-specific exercise therapies, including Schroth-based interventions or other curve-specific corrective programs, unless the independent effect of a non-specific exercise component could be isolated;
7. studies in which non-specific exercise therapy was combined with brace treatment, scoliosis-specific exercise therapy, or another active intervention, when the independent effect of the non-specific exercise component could not be isolated.

For quantitative synthesis, intervention nodes were assigned according to predefined clinical criteria. Interventions primarily targeting trunk stabilization and deep core muscle control were classified as core muscle group training. Interventions primarily targeting proprioception, balance, postural control, or task-oriented motor control were classified as proprioceptive training. Interventions mainly involving stretching, spinal mobility, yoga, or Pilates were classified as spinal flexibility training. Interventions combining general breathing, flexibility, trunk-control, and corrective gymnastics without formal curve-specific three-dimensional correction were classified as general corrective exercise. The detailed rationale for assigning each intervention to a network node is provided in Supplementary Table S3.

### 2.3 Screening and data extraction

All retrieved records were imported into EndNote for automatic and manual deduplication. Two independent reviewers then screened the studies based on the predefined inclusion and exclusion criteria. The screening procedure consisted of two stages: (1) title and abstract screening to remove clearly irrelevant studies, followed by (2) full-text assessment to determine final eligibility. Any discrepancies were resolved through discussion or adjudication by a third reviewer.

Data extraction was independently conducted by two reviewers using a standardized form. Extracted information included general study characteristics, participant details, intervention characteristics, and outcome measures. The main characteristics of the included studies are summarized in Table 1.

**Table 1:** Intervention effects of Non-specific exercise therapy.

Author (Year)	Intervention	Participant Age (years)	Intervention Duration (weeks)	Frequency (sessions/ week)	Training Content Summary	Session Duration	Between Sets (s)	Repetitions per Set
Deng et al. (2021) (13)	Core Muscle Group Training	10.16 ± 2.30	12 (3 Stages)	7/14/7	Wall stretches, supine flexion–extension, equipment-assisted training	10–15 s	5	3–8
Ko and Kang (2017) (14)	Core Muscle Group Training	12.71 ± 0.72	12	3	Posterior pelvic tilt, curl-up, quadruped alternating lifts, forward-reaching walk	12 repetitions per exercise	-	3
Kai-le et al. (2024) (15)	Core Muscle Group Training	12.1 ± 1.8	10	3	Double bridge, single bridge, reverse bridge, equipment-assisted exercises	10–15s	-	10
Liu and F (2024) (16)	Core Muscle Group Training	12.59 ± 2.85	12	3	Plank, dynamic repetitions, unstable surface training, equipment-assisted	30s	30	3–5
Fanling et al. (2020) (17)	Core Muscle Group Training	14.9 ± 2.0	12 (3 Stages)	7/14/7	Static holds, abdominal bridge, quadruped support, trunk rotation, crawling exercises	8–30s	2–3	3–8
Monticone et al. (2014) (18)	Proprioceptive and Task-Oriented Training	12.4 ± 1.1	12	2	Active postural correction, task-oriented practice, turning exercises	30min	-	1
Nie et al. (2025) (19)	PNF/SPS/Combined Training	13.5–14.25	12	3	PNF combined with spiral muscle chain training to enhance coordination and balance	60min	-	1
Liu et al. (2021) (20)	Yoga Training	18.34 ± 0.89	12	4	Stretching, core activation, meditation, and relaxation	90min	-	2–4
Fishman (2021) (21)	Yoga-based isometric exercise	14.0 ± 2.5	60	1	Side plank, half-moon pose, elevated side plank	held to maximum endurance	-	-
Manzak Dursun et al. (2024) (22)	Pilates Training	10∼18	12	7	Basic Pilates training (hybrid remote and home-based)	60min	-	-
Xavier et al. (2020) (23)	Aerobic and Resistance Training	16 ± 2	12	3	Running, cycling, and upper/lower limb resistance training	40 + 10min	30	1–2
Lau et al. (2021) (24)	HIIT (E-Fit)	12.8 ± 0.9	42	5	Home-based high-intensity interval training	7min	-	-
Liu et al. (2023) (25)	General corrective exercise	11.52 ± 0.50	12	6	Breathing, flexibility, and core combination exercises	30–60min	-	2
Fu et al. (2018) (26)	General corrective exercise	12–13	12	10 + 5	Stretching and strengthening combination exercises	-	-	-

The intervention frequency in the table refers to the number of sessions per week. “PNF” denotes proprioceptive neuromuscular facilitation, and “SPS” refers to spiral stabilization training. Pilates and yoga interventions are represented by typical posture examples. Some studies implemented a phased progression design, with training content advancing stepwise by stage.

### 2.4 Risk-of-bias assessment

Randomized controlled trials were assessed using the revised Cochrane risk-of-bias tool for randomized trials, version 2 (RoB 2), which evaluates bias arising from the randomization process, deviations from intended interventions, missing outcome data, measurement of the outcome, and selection of the reported result. Non-randomized controlled studies were assessed separately using ROBINS-I, which evaluates bias due to confounding, selection of participants, classification of interventions, deviations from intended interventions, missing data, measurement of outcomes, and selection of the reported result. The risk-of-bias results for randomized and non-randomized studies are reported separately. Because the primary objective of the risk-of-bias assessment was to evaluate the internal validity of evidence contributing to the Cobb angle quantitative synthesis and network estimates, formal RoB 2 and ROBINS-I assessments were restricted to studies included in the Cobb angle quantitative synthesis. Studies retained only in the qualitative synthesis did not provide extractable Cobb angle data suitable for quantitative analysis and therefore did not contribute to the pairwise meta-analysis, network meta-analysis, league table, SUCRA ranking, or CINeMA assessment. These studies were used only as contextual evidence and were not included in Figure 2.

### 2.5 Statistical analysis

Network meta-analysis was conducted using Stata version 18.0 with the network suite of commands and the mvmeta framework for model fitting, network estimation, ranking, and graphical presentation. A contrast-based frequentist network meta-analysis was performed under the consistency assumption. Continuous outcomes were summarized as mean differences (MDs) with 95% confidence intervals (CIs). Routine care/exercise was used as the reference intervention.

Given the clinical and methodological heterogeneity across exercise protocols, a random-effects consistency model was used as the primary model, assuming a common between-study heterogeneity variance across treatment comparisons. Between-study variance was estimated using restricted maximum likelihood. Fixed-effect analyses were performed only as sensitivity analyses where applicable.

For multi-arm studies, correlations induced by shared comparator groups were handled within the multivariate meta-analysis framework. If variance data were zero, missing, or could not be derived from the reported information, the original article and supplementary materials were checked. Studies without extractable variance data were excluded from quantitative synthesis and retained only in qualitative synthesis.

Because the final evidence network did not contain closed loops, formal inconsistency testing using loop-specific or design-by-treatment interaction approaches was not applicable. Therefore, the consistency assumption could not be empirically tested and was interpreted together with the transitivity assessment. Transitivity was assessed by comparing potential effect modifiers across intervention nodes.

The overall pairwise meta-analysis was calculated using Cobb angle endpoint means and SDs extracted from each study arm. Endpoint values included post-intervention, end-of-treatment, or follow-up Cobb angle measurements as reported in the original studies. The MD was calculated as the endpoint mean in the exercise group minus the endpoint mean in the comparator group; therefore, negative MDs favored the exercise intervention. Change-from-baseline scores and adjusted estimates were not used in the primary quantitative synthesis unless explicitly stated. The analytic dataset is shown in Supplementary Table S4A, and the outcome definition, time point, source location, and SD extraction or conversion method for each study are provided in Supplementary Table S4B.

#### 2.5.1 Sensitivity analysis

Sensitivity analyses were conducted to examine the robustness of the findings. First, a strict RCT-only sensitivity analysis was performed by excluding all non-randomized controlled studies, even if this resulted in the disappearance of some intervention nodes. Second, the primary mixed-design analysis including both randomized and non-randomized controlled studies was compared with the RCT-only analysis to assess the influence of study design on network structure and treatment rankings. Third, studies judged as having serious or critical risk of bias were excluded to examine the influence of high-risk studies. When a sensitivity analysis resulted in a sparse or disconnected network, the findings were summarized using direct pairwise evidence or descriptive comparison rather than full network ranking.

An additional classification-based sensitivity analysis was conducted by excluding interventions with low or moderate classification certainty to examine whether borderline intervention classification influenced the network structure and treatment rankings.

A brace-exposure sensitivity analysis was conducted by excluding studies included in the Cobb angle quantitative synthesis that reported brace co-intervention or unclear group-specific brace exposure. Studies retained only in qualitative synthesis were not included in this sensitivity analysis because they did not contribute to the Cobb angle quantitative synthesis. When the exclusion of brace-exposure studies resulted in a sparse network, results were summarized as a robustness check rather than interpreted as definitive rankings. Details on brace exposure, group-specific brace proportions, matching of brace protocols, and sensitivity-analysis handling are provided in Supplementary Table S6.

#### 2.5.2 Assessment of confidence in network evidence

CINeMA assessment was performed independently by two reviewers, and disagreements were resolved through discussion. The equivalence range for Cobb angle was set at ±5°, based on commonly used thresholds for Cobb angle measurement variability and curve progression. Imprecision and heterogeneity were judged according to whether the confidence interval or prediction interval crossed this equivalence range and whether the result could lead to different clinical interpretations.

Because the network had a star-shaped structure without closed loops, empirical assessment of incoherence by direct–indirect comparison was not possible. Therefore, the incoherence domain was interpreted as not directly estimable rather than as evidence of detected inconsistency. Any concern in this domain reflected uncertainty caused by the inability to empirically verify consistency in the sparse network. All CINeMA judgments were based on the final corrected Cobb angle dataset used for the primary network meta-analysis.

## 3 Results

### 3.1 Search outcomes

A total of 3,373 relevant articles were initially identified through systematic database searches and stepwise screening. After removing duplicates and excluding records according to predefined eligibility criteria, 14 studies were ultimately included (13–26) The detailed selection process is presented in Figure 1.

**Figure 1:**
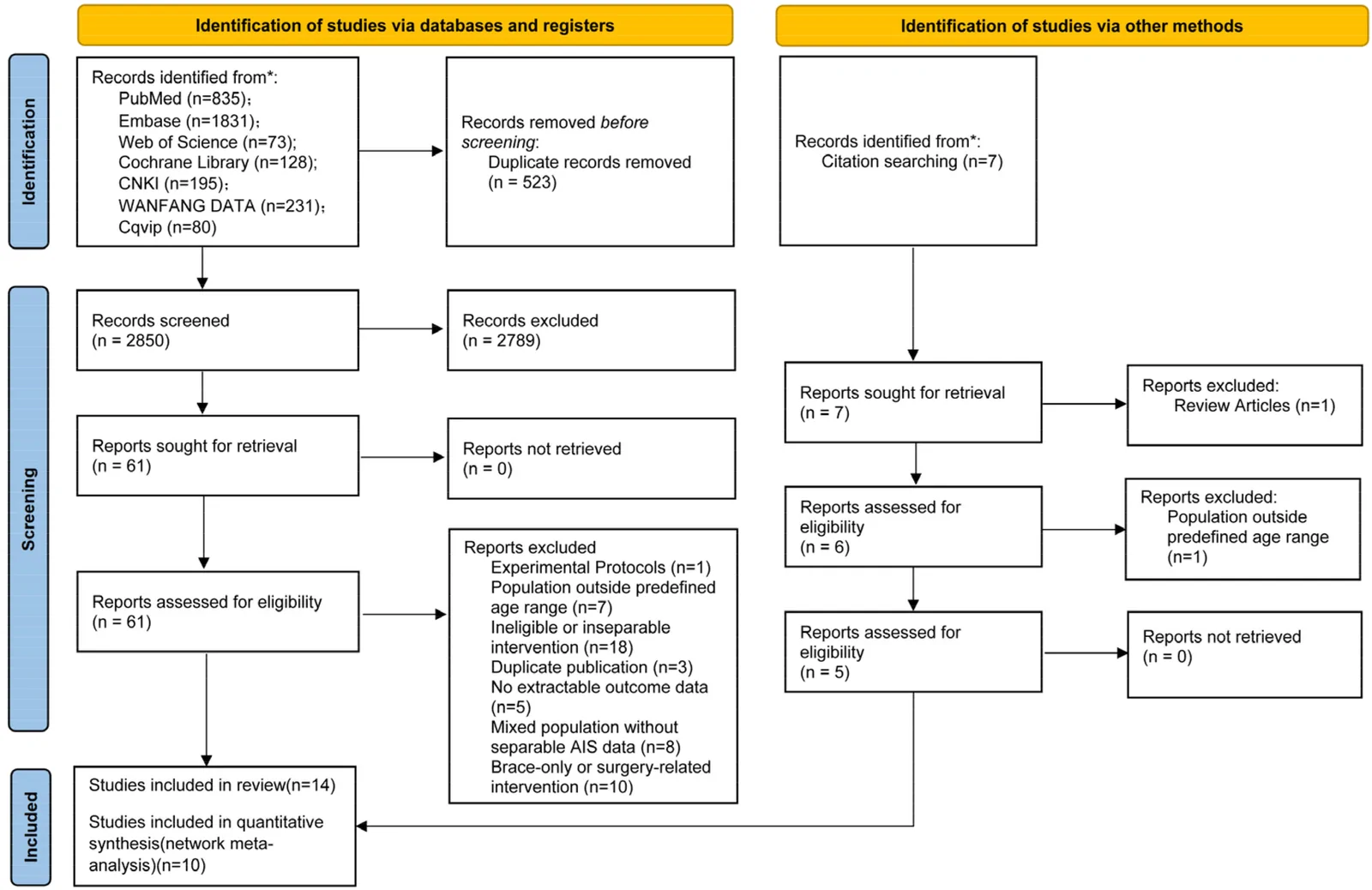
Study selection process.

### 3.2 Basic characteristics of included studies

This review included 14 studies (6 in English and 8 in Chinese) involving a total of 723 participants—384 in the experimental groups and 339 in the control groups (Table 1). Among them, 10 studies involving 589 participants provided extractable Cobb angle data suitable for quantitative synthesis and were included in both the pairwise meta-analysis and the primary network meta-analysis. The remaining four studies were retained in the qualitative synthesis but were not included in the Cobb angle quantitative synthesis because extractable Cobb angle data were unavailable or incompatible with the final analysis. The study-level analytic dataset and sample sizes used in each analysis are provided in Supplementary Table S4A. The outcome definition, time point, source location, and SD extraction or conversion method for Cobb angle data are provided in Supplementary Table S4B.

### 3.3 Risk-of-bias assessment for studies included in the cobb angle quantitative synthesis

Risk-of-bias assessment was performed for the 10 studies that contributed data to the Cobb angle quantitative synthesis. These included five randomized controlled trials assessed using RoB 2 and five non-randomized controlled studies assessed using ROBINS-I. The results are summarized in Figure 2. The remaining four studies were retained only in the qualitative synthesis and did not contribute effect estimates to the Cobb angle pairwise meta-analysis, network meta-analysis, SUCRA ranking, league table, or CINeMA assessment; therefore, they were not included in the risk-of-bias figure. For randomized controlled trials assessed using RoB 2, two studies were judged as having some concerns overall, and three studies were judged as having a high overall risk of bias. The main concerns were related to the randomization process, measurement of the outcome, and selection of the reported result. In particular, several studies provided insufficient information on allocation procedures, and some studies were judged to have high risk in outcome measurement, partly because blinding of outcome assessors and standardized Cobb angle measurement procedures were insufficiently reported. Therefore, the randomized evidence should be interpreted cautiously.

**Figure 2:**
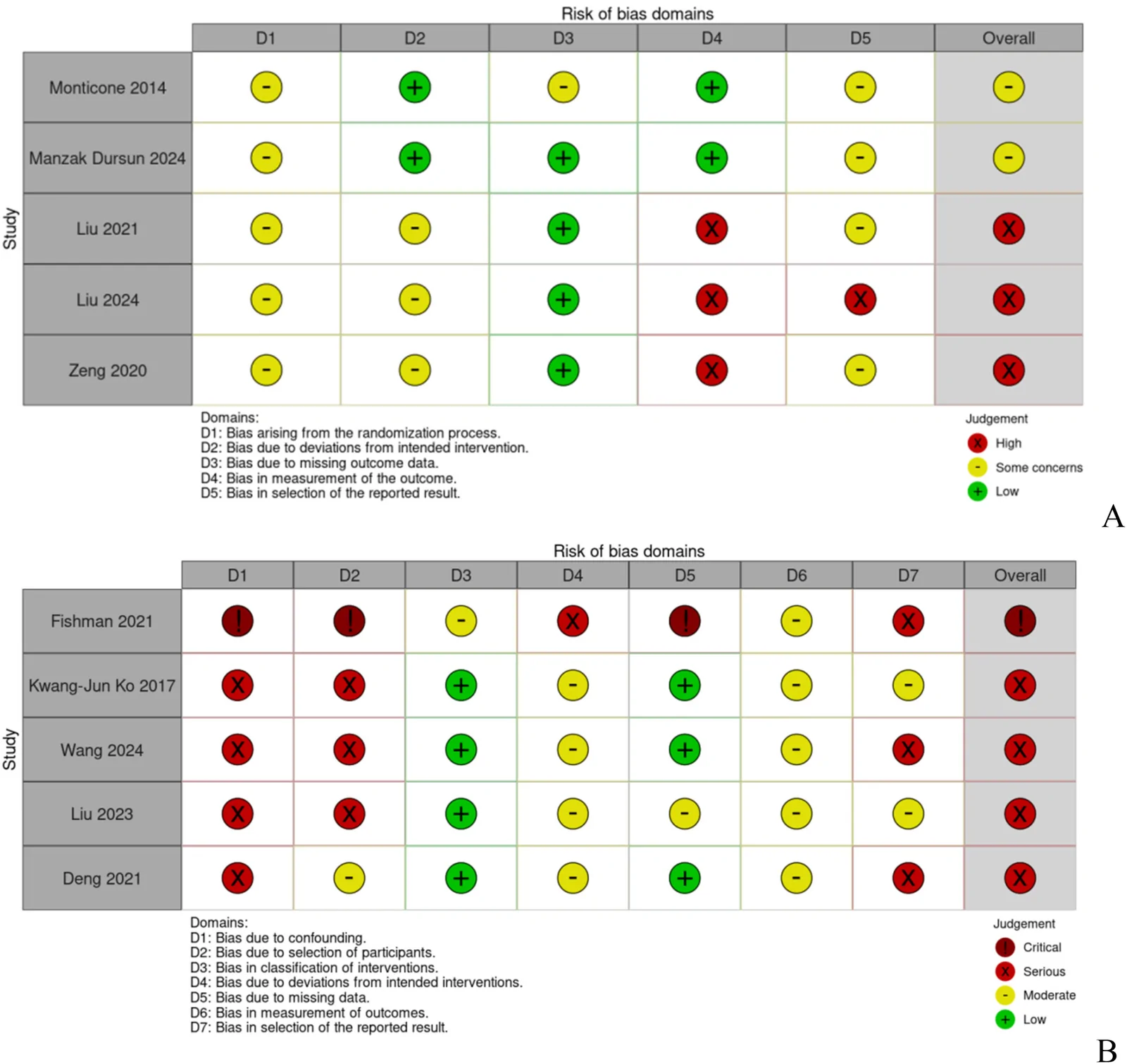
Risk-of-bias assessment of studies included in the cobb angle quantitative synthesis. **(A)** Risk-of-bias assessment for randomized controlled trials using RoB 2. **(B)** Risk-of-bias assessment for non-randomized controlled studies using ROBINS-I.

For non-randomized controlled studies assessed using ROBINS-I, one study was judged as having a critical overall risk of bias, and four studies were judged as having a serious overall risk of bias. The main concerns were bias due to confounding and bias due to selection of participants. Additional concerns were identified in deviations from intended interventions, missing data, measurement of outcomes, and selection of the reported result. Overall, the non-randomized evidence had important methodological limitations and was interpreted cautiously in the synthesis.

Studies retained only in the qualitative synthesis were not included in this figure because they did not contribute effect estimates to the Cobb angle quantitative synthesis.

## 4 Results of the Cobb angle network meta-analysis

### 4.1 Primary network meta-analysis of Cobb angle

The primary network meta-analysis included 10 controlled studies with extractable Cobb angle data and evaluated five intervention nodes: core muscle group training, spinal flexibility training, proprioceptive training, general corrective exercise, and routine care/exercise as the common comparator. The network showed a star-shaped structure centered on routine care/exercise, with no direct head-to-head comparisons between active exercise interventions and no closed loops; therefore, formal inconsistency testing was not applicable (Figure 3a). Potential effect modifiers were compared separately to assess transitivity.

**Figure 3:**
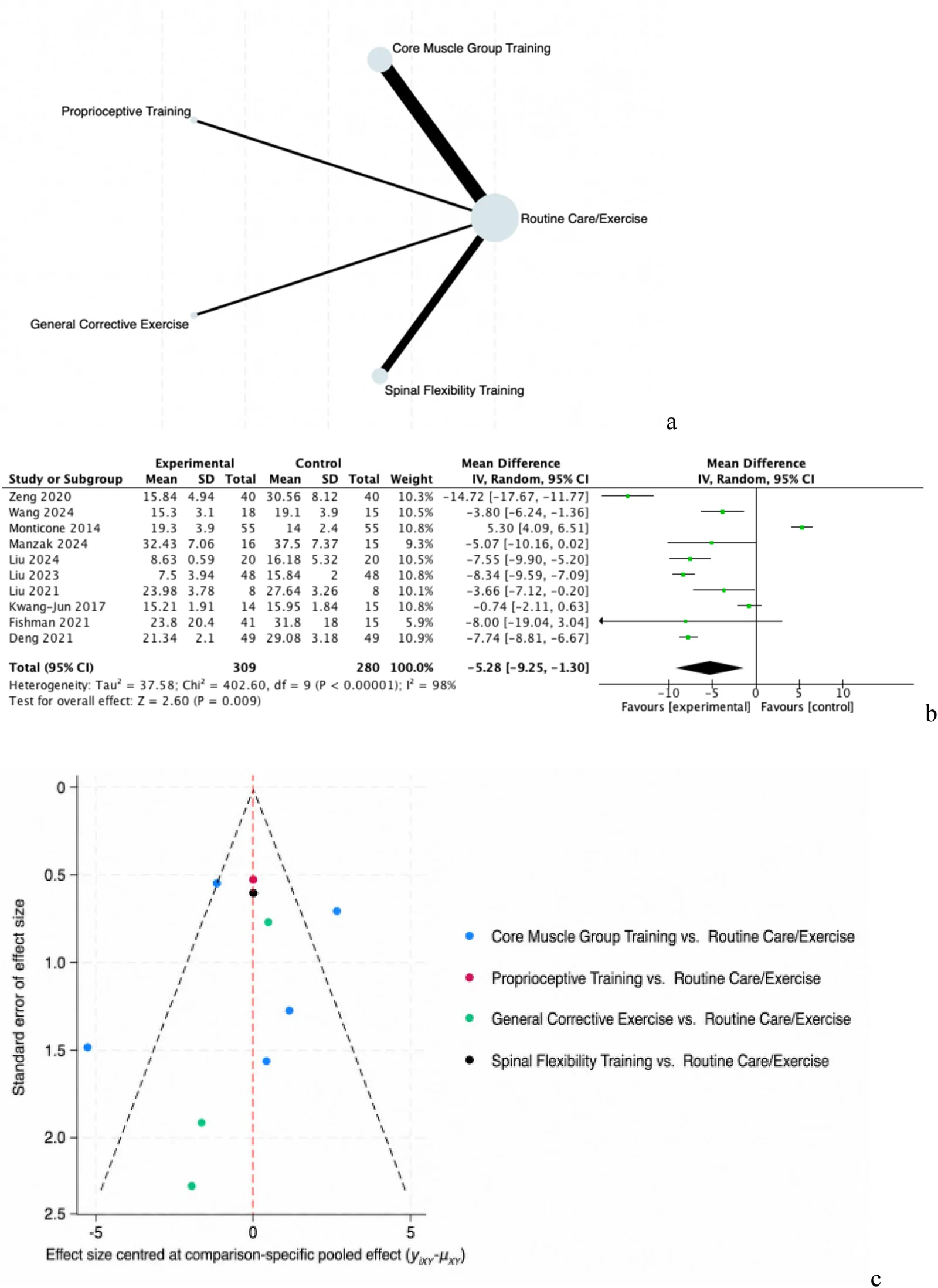

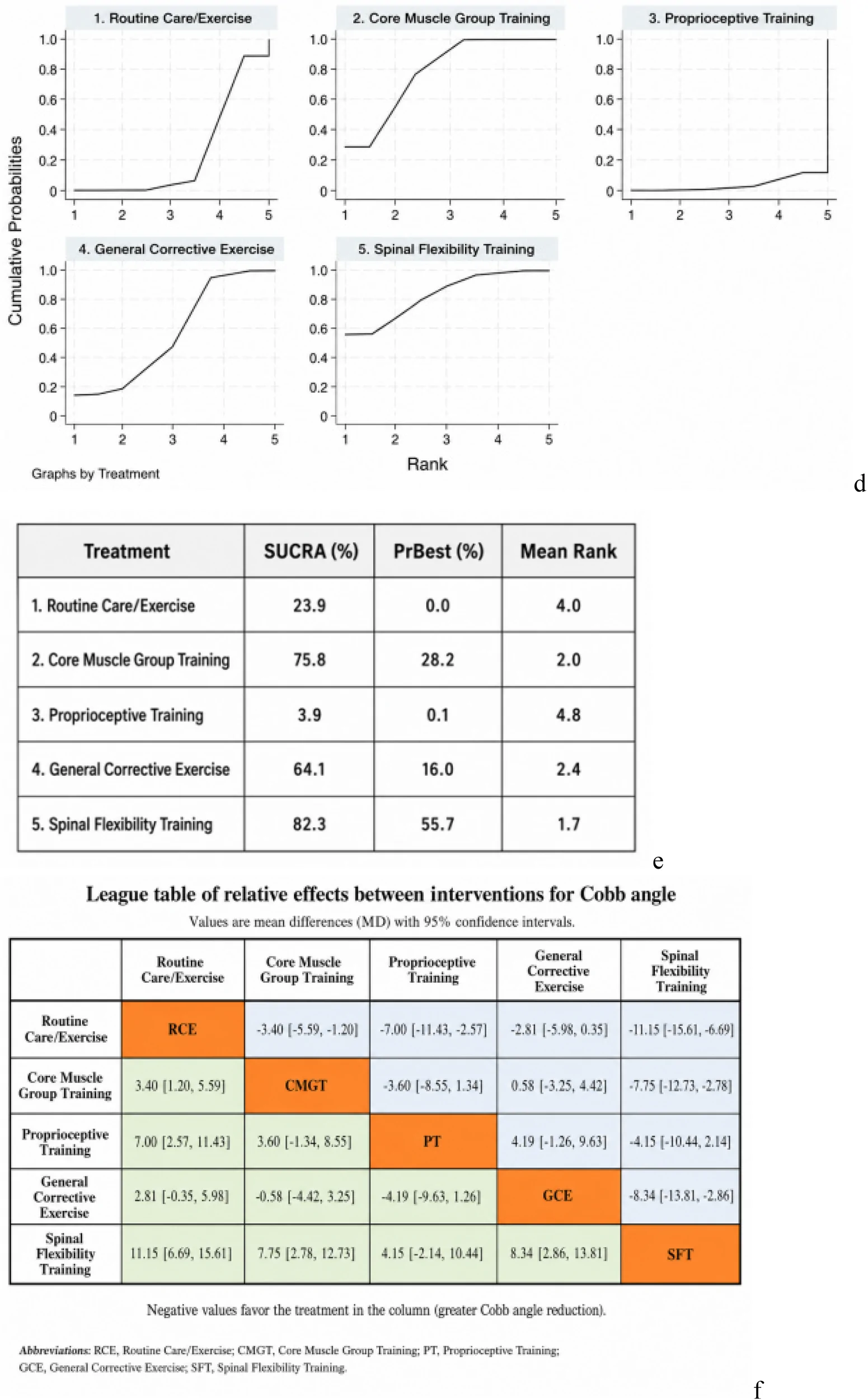
Network meta-analysis results for cobb angle. **(a)** Network plot; **(b)** overall pairwise forest plot; **(c)** comparison-adjusted funnel plot; **(d)** cumulative ranking probability curves; **(e)** SUCRA ranking table; and **(f)** league table of relative effects. MDs less than zero favor exercise interventions in panel b. In panel f, negative values favor the treatment in the column. CI, confidence interval; MD, mean difference; SUCRA, surface under the cumulative ranking curve. The prediction interval for the overall pairwise random-effects estimate was calculated to reflect between-study heterogeneity and should be interpreted as exploratory.

The overall pairwise random-effects meta-analysis suggested that non-specific exercise therapies were associated with a greater reduction in Cobb angle than control conditions (MD = −5.28, 95% CI −9.25 to −1.30; 95% prediction interval: −20.18 to 9.62; Figure 3b). However, substantial heterogeneity was observed (I^2^ = 98%, *P* < 0.00001), and the prediction interval was wide and crossed the null value. Therefore, this pooled estimate should be interpreted as an exploratory summary of the overall direction of direct evidence rather than as a definitive estimate of the effect of non-specific exercise therapy as a single homogeneous intervention.

The comparison-adjusted funnel plot suggested potential asymmetry, indicating possible small-study effects or publication bias; however, because the number of studies was limited, this finding should be considered exploratory (Figure 3c). SUCRA analysis suggested that spinal flexibility training had the highest ranking probability (SUCRA = 82.3%, PrBest = 55.7%), followed by core muscle group training (SUCRA = 75.8%) and general corrective exercise (SUCRA = 64.1%). Proprioceptive training had the lowest SUCRA value (3.9%) (Figures 3d,e). The league table showed that several non-specific exercise interventions had favorable estimates compared with routine care/exercise, but comparisons between active interventions were generally imprecise and often crossed the null value (Figure 3f). Therefore, the relative superiority of one active intervention over another remains uncertain.

Pairwise meta-analysis was conducted to summarize the direct evidence for each intervention category versus routine care/exercise. Overall, most pooled direct estimates showed a favorable direction for non-specific exercise therapies in Cobb angle reduction, but the magnitude and precision of the effects varied across intervention categories and individual studies (Figure 4). Core muscle group training was supported by the largest number of studies and generally showed favorable effects compared with routine care/exercise, although the effect sizes varied between studies. Spinal flexibility training was supported by three studies and also showed a favorable direction of effect, but the pooled estimate was imprecise, with a relatively wide confidence interval. General corrective exercise was supported by only one study and showed a favorable direction of effect, but the limited evidence and wide confidence interval indicate substantial uncertainty. Proprioceptive training was also supported by only one study, and its estimate was uncertain. Therefore, although the direct evidence generally suggested potential benefits of exercise-based interventions, these pairwise estimates should be interpreted cautiously because of the small number of studies in several comparisons, wide confidence intervals, and variability across intervention protocols.

**Figure 4:**
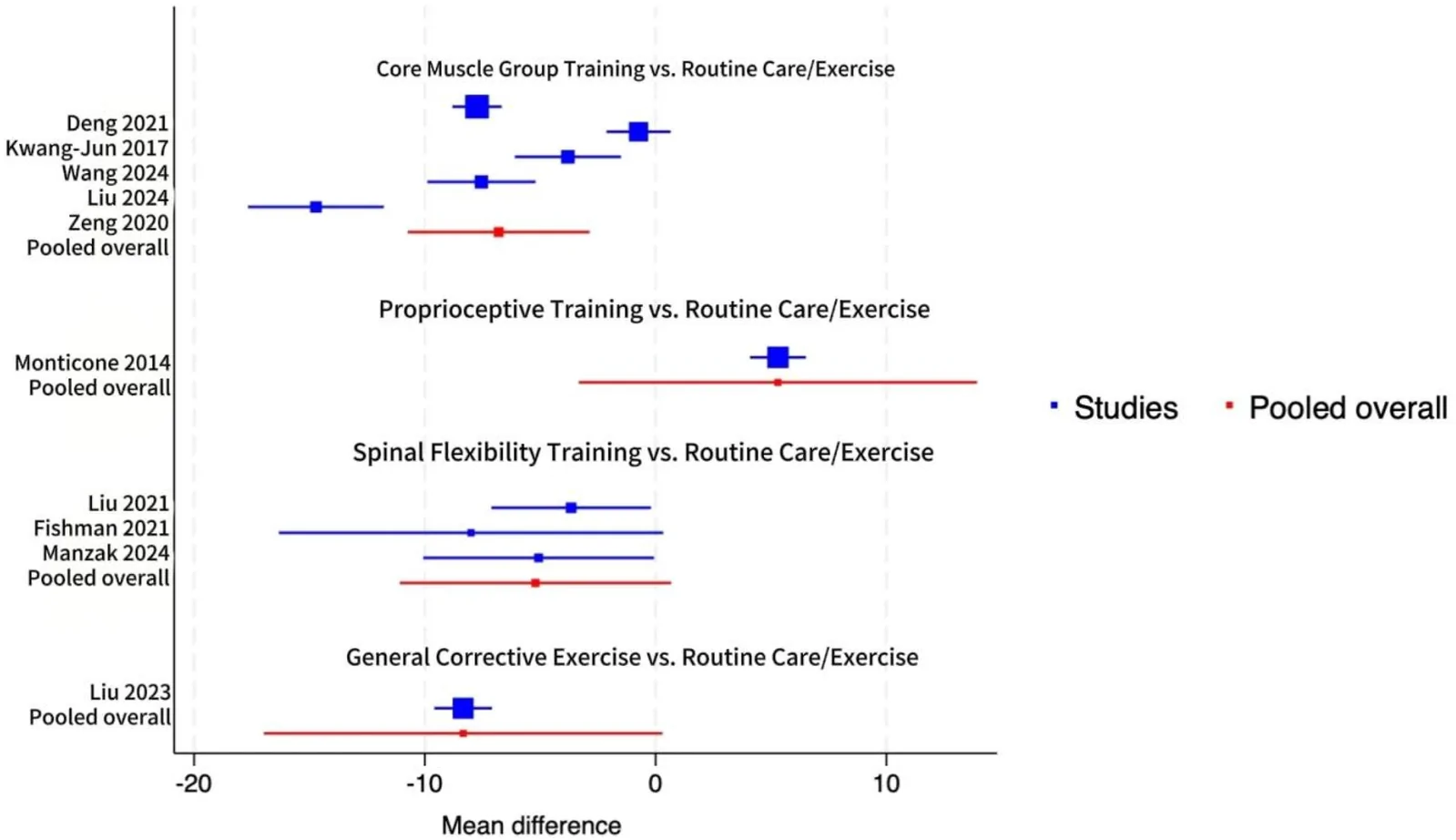
Direct pairwise evidence for each non-specific exercise category versus routine care/exercise for cobb angle. Blue squares represent individual study estimates, and red squares represent pooled direct estimates within each intervention category. Mean differences less than zero favor the exercise intervention. Accordingly, interpretation of treatment effects was based primarily on category-specific direct estimates and network estimates, whereas the overall pairwise estimate was treated only as a descriptive and exploratory summary.

This overall pairwise estimate should not be interpreted as the treatment effect of any specific intervention node, because it pooled clinically different non-specific exercise categories and comparator conditions.

### 4.2 Assessment of transitivity

Transitivity was assessed by comparing potential effect modifiers across intervention nodes, including participant age, baseline Cobb angle, skeletal maturity, curve type, intervention duration, training frequency, session duration, supervision, brace co-intervention, and comparator condition. The actual ranges and distributions of these potential effect modifiers are shown in Table 2. Participant age was broadly comparable across nodes. However, baseline Cobb angle, skeletal maturity reporting, curve type, intervention dose, supervision, brace use, and comparator condition differed across studies.

**Table 2:** Distribution of potential effect modifiers across intervention nodes for transitivity assessment.

Potential effect modifier	Core Muscle Group Training	Proprioceptive Training	Spinal Flexibility Training	General Corrective Exercise
Number of studies	5	1	3	1
Participant age, years	10.1–14.9	12.5 ± 1.1	14.0–18.3	11.52 ± 0.50
Baseline Cobb angle	14.5–30.6	19.3 ± 3.9	20.5–40.3	15.84 ± 2.00
Skeletal maturity	NR in all studies	Risser 0–1; all <2 at baseline	Mean Risser 2.75–2.9; NR in 1 study	NR
Curve type	Mixed; mostly incompletely reported	Thoracic, lumbar, thoracolumbar, and S-shaped	Mixed thoracic/ lumbar/ thoracolumbar/ double curves	Mild AIS; curve pattern NR
Intervention duration	10–13 weeks; NR in 1 study	Until skeletal maturity; mean 42.8 ± 9.1 months	12 weeks to ≥ 5 months	12 weeks
Frequency	3 sessions/week to 2 sessions/day; NR in 1 study	1 outpatient + 2 home sessions/week	4–7 sessions/week; daily practice recommended in 1 study	Home training 2 sessions/day; group training 1 session/week
Session duration	20–60 min; NR in 1 study	60 min outpatient; 30 min home	<5–90 min	30 min home; 60 min group
Supervision	Fully supervised in 1 study; unclear/partial in 4 studies	Supervised outpatient sessions; home diary checked weekly	Mixed: self-practice, home-based, or hybrid supervised	Parent-supervised home training + weekly supervised group training
Brace co-intervention	Used in 1 study; not reported in 4 studies	NR; no brace described	None in 1 study; used in ∼50% in 1 study; NR in 1 study	NR; no brace use was described
Comparator condition	Heterogeneous: usual care, no structured exercise, brace/usual care, traditional exercise, or massage	Matched traditional general exercise programme	No specific exercise, low-adherence yoga, or less-supervised Pilates	Health education + observation

The most important potential effect modifiers were considered to be baseline curve severity, skeletal maturity, curve type, intervention dose, and comparator condition. Baseline Cobb angle may modify treatment response because mild and moderate curves may respond differently to conservative exercise. Skeletal maturity is also important because curve progression risk differs before and after peak growth. However, skeletal maturity and curve type were incompletely reported in several studies, which limited the assessment of transitivity. Comparator conditions also varied, including no intervention, routine care, physical education, conventional exercise, and massage. Therefore, the transitivity assumption was considered uncertain rather than fully established, and the network estimates and SUCRA rankings should be interpreted cautiously.

Brace co-intervention was audited separately because brace treatment may modify Cobb angle outcomes. DENG LD 2021 reported routine brace treatment and nursing in both groups, with additional core muscle strength training in the intervention group. Manzak 2024 reported similar brace use between groups, with 8/16 participants in the training group and 7/15 participants in the control group using a brace. Lau 2021 also reported brace use, but this study was retained only in qualitative synthesis and did not contribute to the Cobb angle quantitative synthesis. Details are provided in Supplementary Table S6.

### 4.3 Sensitivity analysis according to study design

A strict RCT-only sensitivity analysis was conducted by excluding all non-randomized controlled studies. After exclusion, five randomized controlled trials remained, and the general corrective exercise node disappeared because it was supported only by non-randomized evidence. The RCT-only network included four nodes: routine care/exercise, core muscle group training, proprioceptive training, and spinal flexibility training. The full results are provided in Supplementary Figure S1.

In the RCT-only sensitivity analysis, core muscle group training ranked highest (SUCRA = 98.7%, PrBest = 96.2%, mean rank = 1.0), followed by spinal flexibility training (SUCRA = 65.0%, PrBest = 3.8%, mean rank = 2.1), routine care/exercise (SUCRA = 33.4%, mean rank = 3.0), and proprioceptive training (SUCRA = 3.0%, mean rank = 3.9).

Compared with the primary mixed-design analysis, the RCT-only analysis changed the network structure and removed the general corrective exercise node. Core muscle group training ranked highest in the RCT-only analysis, whereas spinal flexibility training ranked highest in the primary analysis. These findings indicate that treatment rankings were sensitive to study design and network structure. Therefore, SUCRA rankings should be interpreted as exploratory rather than definitive evidence of superiority.

Because all non-randomized controlled studies were judged as having serious or critical risk of bias according to ROBINS-I, excluding studies with serious or critical risk of bias resulted in the removal of all non-randomized studies. Therefore, the resulting dataset was the same as the strict RCT-only sensitivity analysis. This further indicates that the primary mixed-design rankings were influenced by the inclusion of non-randomized studies with important methodological limitations.

A classification-based sensitivity analysis was also conducted by excluding interventions with low or moderate classification certainty. After exclusion, the evidence network became sparse and several intervention nodes were no longer represented. Therefore, a complete network ranking was not considered reliable. This finding suggests that the primary network structure and treatment rankings were partly influenced by interventions with uncertain classification, and the rankings should therefore be interpreted cautiously. In the eligibility/classification sensitivity analysis, studies with borderline eligibility or moderate-to-low classification certainty were excluded. After their exclusion, the evidence network became sparse and several comparisons were no longer estimable; therefore, complete network ranking was not considered reliable. This finding further supports cautious interpretation of the primary network rankings.

A brace-exposure sensitivity analysis was conducted by excluding studies in the Cobb angle quantitative synthesis with reported brace co-intervention or unclear group-specific brace exposure. DENG LD 2021 and Manzak 2024 were excluded from this sensitivity-analysis dataset. Lau 2021 was not considered in this analysis because it was retained only in qualitative synthesis and did not contribute to the Cobb angle quantitative synthesis. After exclusion, eight studies remained, involving 244 participants in the experimental groups and 216 participants in the comparator groups, with a total of 460 participants. The network retained all primary intervention nodes but became sparser. Details of brace exposure and sensitivity-analysis handling are provided in Supplementary Table S6. Therefore, this sensitivity analysis was interpreted as a robustness check rather than as definitive evidence for treatment ranking.

### 4.4 Confidence in network evidence

The confidence in the network estimates for Cobb angle was assessed using CINeMA (Figure 5). Overall, the certainty of the network evidence was limited. The comparison between core muscle group training and routine care/exercise was rated as very low confidence, mainly because of concerns related to within-study bias, reporting bias, imprecision, and incoherence. The comparison between proprioceptive training and routine care/exercise was also rated as very low confidence because of reporting bias, imprecision, and incoherence. General corrective exercise versus routine care/exercise was rated as low confidence, mainly because of reporting bias, indirectness, imprecision, and incoherence. Routine care/exercise versus spinal flexibility training was rated as moderate confidence, with downgrading mainly due to reporting bias, heterogeneity, and incoherence. Because no closed loops were available, incoherence could not be empirically estimated by comparing direct and indirect evidence. Therefore, concerns in the incoherence domain were interpreted as uncertainty arising from the inability to verify consistency in this sparse network, rather than as detected statistical inconsistency.

**Figure 5:**
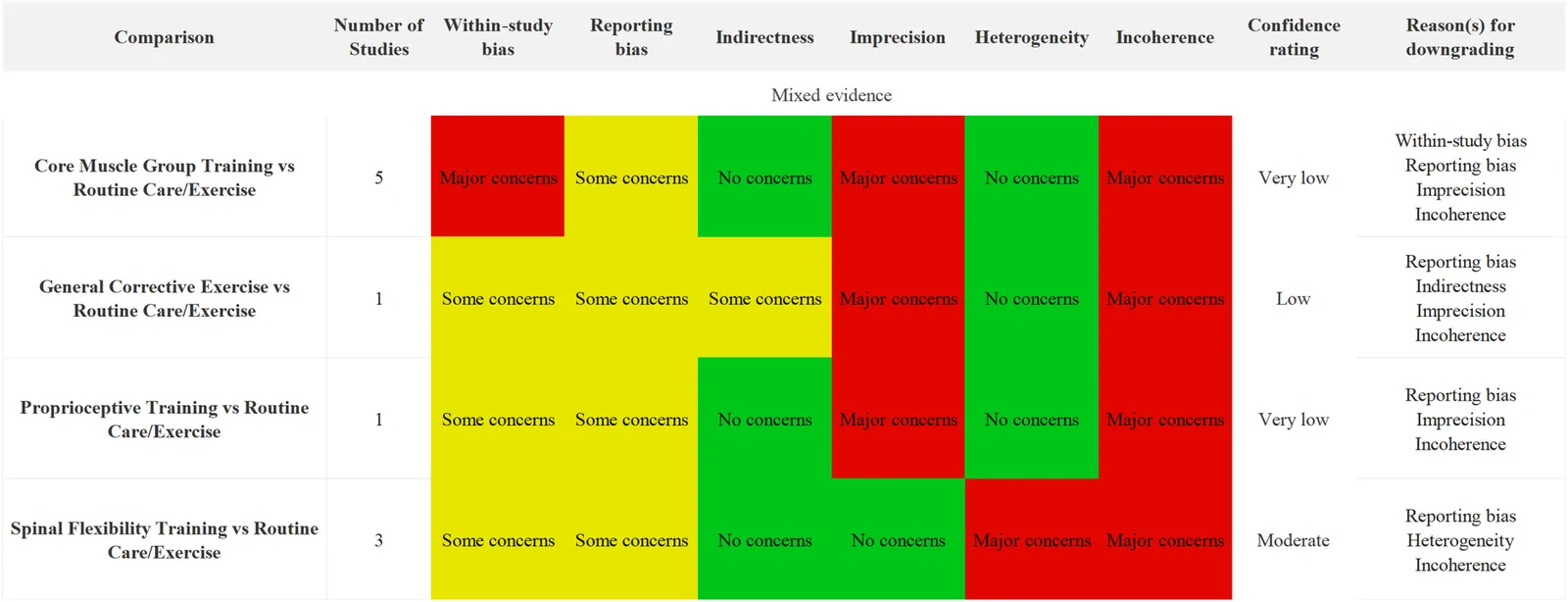
CINeMA assessment of confidence in network meta-analysis estimates for the main active-versus-control comparisons. The confidence in each network comparison was evaluated across six domains: within-study bias, reporting bias, indirectness, imprecision, heterogeneity, and incoherence. Because the evidence network had a star-shaped structure and contained no closed loops, incoherence could not be empirically assessed by comparing direct and indirect evidence. In the CINeMA implementation used in this review, a “not estimable” category was not available; therefore, “major concerns” for incoherence reflects the absence of an empirical incoherence assessment rather than evidence of detected inconsistency. The CINeMA assessment for all 10 network comparisons is provided in Supplementary Table S5.

These findings indicate that the comparative network estimates should be interpreted cautiously. Although some interventions showed favorable effect estimates or higher ranking probabilities, the limited certainty of the evidence suggests that the ranking results should be regarded as exploratory rather than definitive evidence of superiority or firm clinical recommendations.

## 5 Discussion

### 5.1 Evaluation of the efficacy and translational value of non-specific exercise therapies

This systematic review and network meta-analysis compared the effects of several non-specific exercise modalities for AIS. The pairwise random-effects meta-analysis suggested that non-specific exercise therapies may be associated with reductions in Cobb angle compared with control conditions. In the primary network analysis, spinal flexibility training and core muscle group training appeared to have higher ranking probabilities than the other intervention categories. However, these findings should be interpreted cautiously because of substantial heterogeneity, sparse network structure, small sample sizes, concerns regarding transitivity, limited confidence in network evidence, and the inclusion of both randomized and non-randomized controlled studies.

The study-level effect estimates used in the Cobb angle quantitative synthesis are summarized in Table 3. All values in Table 3 represent Cobb angle endpoint means and SDs used in the primary quantitative synthesis, rather than change-from-baseline scores or adjusted effect estimates. Details on outcome definition, time point, data source location, and SD handling are provided in Supplementary Table S4B. Figure 6 provides a conceptual framework illustrating the potential functional, biomechanical, respiratory, and structural pathways through which non-specific exercise therapies may influence AIS-related outcomes. This figure should be interpreted as a hypothesis-generating framework rather than a direct summary of outcomes confirmed by the present network meta-analysis.

**Table 3:** Study-level effect estimates used in the cobb angle quantitative synthesis.

Author (Year)	Study design	Intervention node	Experimental (n)	Control (n)	Experimental Cobb angle endpoint used in analysis, mean ± SD	Comparator Cobb angle endpoint used in analysis, mean ± SD	MD (95% CI)	Risk-of-bias rating
Deng et al. (2021) (13)	Non-randomized controlled study	Core Muscle Group Training	49	49	21.34 ± 2.10	29.08 ± 3.18	−7.74 (−8.81 to −6.67)	Serious
Ko and Kang (2017) (14)	Non-randomized controlled study	Core Muscle Group Training	14	15	15.21 ± 1.91	15.95 ± 1.84	−0.74 (−2.11 to 0.63)	Serious
Kai-le et al. (2024) (15)	Non-randomized controlled study	Core Muscle Group Training	18	15	15.3 ± 3.1	19.10 ± 3.90	−3.80 (−6.24 to −1.36)	Serious
Fanling et al. (2020) (17)	RCT	Core Muscle Group Training	40	40	15.84 ± 4.94	30.56 ± 8.12	−14.72 (−17.67 to −11.77)	High
Liu and F (2024) (16)	RCT	Core Muscle Group Training	20	20	8.63 ± 0.59	16.18 ± 5.32	−7.55 (−9.90 to −5.20)	High
Monticone et al. (2014) (18)	RCT	Proprioceptive Training	55	55	19.30 ± 3.90	14.00 ± 2.40	5.30 (4.09 to 6.51)	Some concerns
Liu et al. (2021) (20)	RCT	Spinal Flexibility Training	8	8	23.98 ± 3.78	27.64 ± 3.26	−3.66 (−7.12 to −0.20)	High
Fishman (2021) (21)	Non-randomized controlled study	Spinal Flexibility Training	41	15	23.80 ± 20.40	31.80 ± 18.00	−8.00 (−19.04 to 3.04)	Critical
Manzak Dursun et al. (2024) (22)	RCT	Spinal Flexibility Training	16	15	32.43 ± 7.06	37.50 ± 7.37	−5.07 (−10.16 to 0.02)	Some concerns
Liu et al. (2023) (25)	Non-randomized controlled study	General Corrective Exercise	48	48	7.50 ± 3.94	15.84 ± 2.00	−8.34 (−9.59 to −7.09)	Serious

MD, mean difference; CI, confidence interval; RCT, randomized controlled trial; SD, standard deviation. For Cobb angle, lower values indicate better radiographic outcomes. The quantitative synthesis used Cobb angle endpoint values extracted from the corresponding study arms, including post-intervention, end-of-treatment, or follow-up endpoint values as reported in the original studies. The MD was calculated as the experimental-group endpoint mean minus the comparator-group endpoint mean; therefore, negative MDs favor the exercise intervention. Change-from-baseline scores and adjusted effect estimates were not used in the primary quantitative synthesis unless explicitly stated. *P*-values for within-group pre–post changes were not used as evidence of comparative treatment effects. The analytic dataset is shown in Supplementary Table S4A, and details on outcome definition, time point, source location in the original report, and SD extraction or conversion methods are provided in Supplementary Table S4B. Risk-of-bias ratings were based on RoB 2 for randomized controlled trials and ROBINS-I for non-randomized controlled studies.

**Figure 6:**
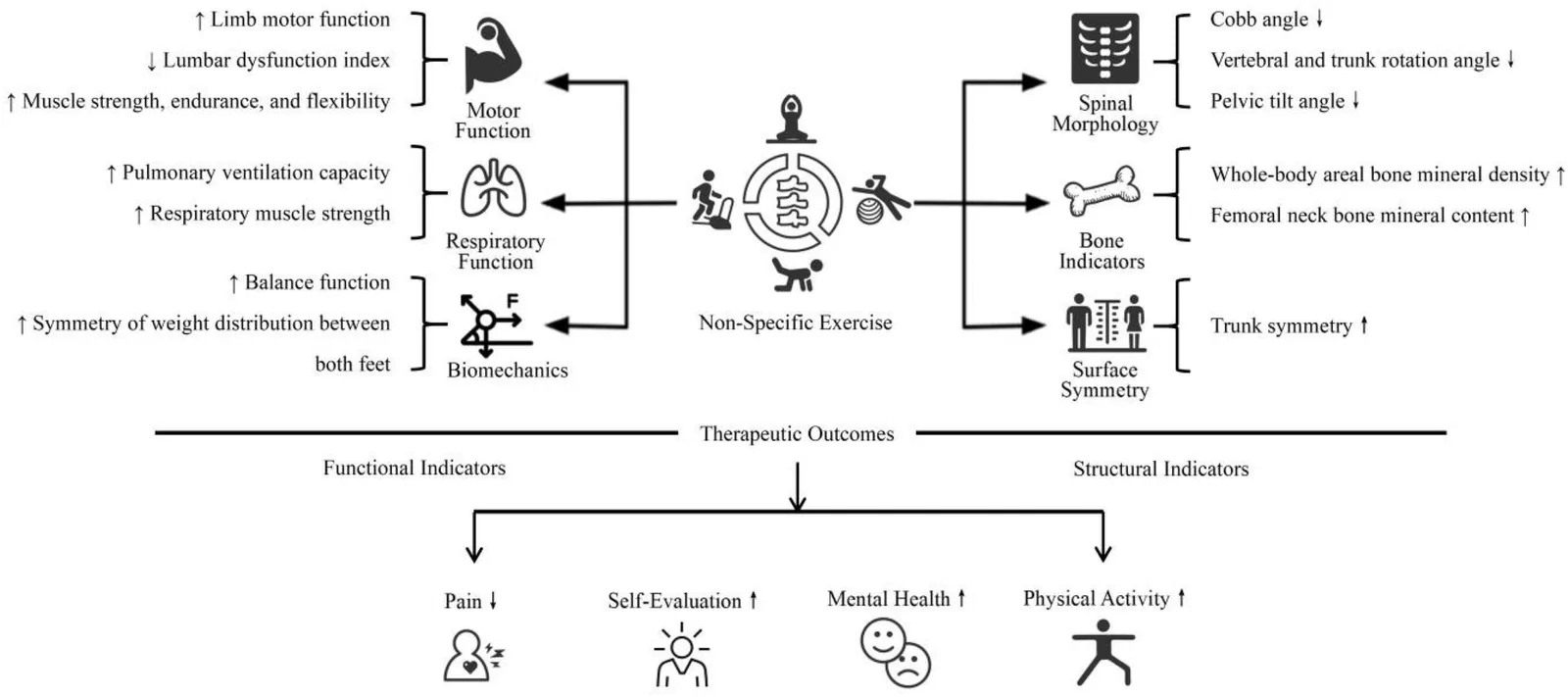
Conceptual framework of potential effects of non-specific exercise therapy in AIS.

Although the pooled reduction in Cobb angle was statistically significant, its clinical relevance should be interpreted cautiously. Cobb angle measurement is affected by observer variability, end-vertebra selection, and measurement method, and a change of approximately 5° is commonly used to distinguish potential true change from measurement error and to classify curve progression (1). However, this threshold is not a formally validated patient-centred minimal clinically important difference. As the pooled mean reduction in the present analysis was approximately 5°, the findings suggest modest short-term radiographic improvement, but whether this represents clinically meaningful structural correction, sustained stabilization, or reduced progression risk remains uncertain.

Non-specific exercise therapies may be more appropriately considered supportive conservative strategies for adolescents with mild-to-moderate AIS, particularly when the goals include posture control, trunk function, physical capacity, adherence, and possibly slowing curve progression.

Across the primary and sensitivity analyses, the relative ranking of individual exercise modalities was not fully stable. In the primary network analysis, spinal flexibility training showed the highest ranking probability, followed by core muscle group training and general corrective exercise. However, in the strict RCT-only sensitivity analysis, the general corrective exercise node disappeared, and core muscle group training ranked highest. This change indicates that the ranking results were influenced by study design and network structure.

Core-focused programs may represent a relatively consistent and clinically plausible intervention component because they aim to enhance recruitment of deep trunk stabilizers, such as the transverse abdominis and multifidus, and improve neuromuscular control. Spinal flexibility-based approaches, including yoga and Pilates, may contribute through improving spinal mobility, reducing soft-tissue tension, and enhancing breathing mechanics. General corrective exercise may also provide benefit through combined breathing, flexibility, and trunk-control components. Nevertheless, because the ranking order changed in sensitivity analysis and several nodes were informed by limited evidence, these intervention-specific interpretations should be considered exploratory rather than confirmatory. Aerobic training may provide supportive physiological benefits for adolescents with AIS by improving cardiopulmonary fitness, muscular endurance, and exercise tolerance. Although the included studies did not evaluate aerobic exercise as an independent intervention within the network meta-analysis, several trials reported improvements in respiratory function and overall physical capacity following aerobic-based programs. These adaptations may indirectly facilitate the performance of corrective exercises or enhance functional outcomes.

However, current evidence is limited and heterogeneous, and the direct effect of aerobic training on spinal curvature remains unclear. More rigorously designed trials are needed before aerobic exercise can be considered a standalone therapeutic recommendation for AIS.

The classification of exercise interventions is an important methodological issue in AIS research. In the revised analysis, we distinguished non-specific exercise therapies from scoliosis-specific exercise approaches according to whether the intervention was individualized based on curve type or used formal three-dimensional scoliosis correction principles. This distinction is clinically relevant because non-specific exercise programs are generally easier to implement in school, community, or home settings, whereas scoliosis-specific approaches require more specialized assessment and therapist training. Nevertheless, some interventions, such as yoga-based postures or corrective gymnastics, may lie on the boundary between general and scoliosis-specific exercise. Therefore, sensitivity analyses were conducted, and the results should be interpreted with this classification uncertainty in mind.

In addition, the risk-of-bias figure focused on studies contributing to the primary Cobb angle quantitative synthesis. Studies retained only for qualitative synthesis were not included in Figure 2 because they did not contribute effect estimates to the primary outcome analysis. This should be considered when interpreting the overall qualitative evidence base.

Brace co-intervention is another important limitation. Although studies were retained when brace exposure appeared comparable between groups and the exercise component could be interpreted as an incremental intervention on top of background care, brace adherence and wear duration were incompletely reported in the affected studies. Therefore, residual confounding by brace exposure could not be fully excluded, and the Cobb angle findings should not be interpreted as pure effects of exercise independent of all co-interventions. Details of brace exposure and the brace-exposure sensitivity analysis are provided in Supplementary Table S6.

### 5.2 Potential mechanisms of non-specific exercise therapy

Although the etiology and pathogenesis of adolescent idiopathic scoliosis (AIS) remain uncertain, existing evidence suggests that its development may involve multifactorial interactions among genetic predisposition, abnormal skeletal growth, muscle imbalance, and biomechanical loading (27). Non-specific exercise therapies may influence AIS-related outcomes through neuromuscular, biomechanical, respiratory, and metabolic pathways (28). However, the present network meta-analysis primarily evaluated Cobb angle and did not directly test these neuromuscular, biomechanical, respiratory, or metabolic mechanisms. Therefore, the following discussion should be interpreted as a hypothesis-generating explanation based on related literature rather than as mechanistic evidence directly established by this review.

#### 5.2.1 Neuro-muscular control and correction of abnormal force lines

Patients with adolescent idiopathic scoliosis (AIS) frequently exhibit abnormalities in neural drive, delayed muscle activation, and compensatory movement patterns. Non-specific exercise therapy restores neuromuscular function through multiple mechanisms. Core stabilization exercises that alternate between static and dynamic activities (e.g., planks, quadruped contralateral lifts) enhance the nervous system's capacity to recruit and coordinate deep core musculature, thereby improving dynamic spinal stability (13). Proprioceptive training, including proprioceptive neuromuscular facilitation (PNF), activates sensory receptors and optimizes afferent feedback pathways, facilitating the perception and active correction of abnormal posture, and reconstructing proper motor control patterns (17). This neuromuscular optimization provides a foundation for correcting abnormal force vectors and restoring postural symmetry.

From a biomechanical perspective, AIS is characterized by asymmetric vertebral loading: the convex-side muscles are overstretched and weakened, whereas the concave-side muscles are shortened and hypertonic (29). Non-specific exercise therapy directly addresses this imbalance. Core training strengthens both local stabilizers (e.g., transverse abdominis, multifidus) (30) and global muscle groups (e.g., rectus abdominis, erector spinae), reinforcing the muscular “scaffold” around the spine, equalizing bilateral vertebral loading, and slowing curve progression. Corrective gymnastics and spinal flexibility training employ targeted combinations of stretching and strengthening to release soft tissue tension on the concave side, enhance convex-side muscle strength, and gradually restore mechanical equilibrium around the spine. Additionally, aerobic training enhances overall muscular endurance, creating a more stable environment for spinal support (24).

#### 5.2.2 Bone health and metabolic hypotheses

Bone health may be relevant to AIS progression, as some adolescents with AIS present with low-normal bone mineral density during growth (31). Exercise may influence bone mass and bone metabolism in adolescents more broadly (32), and bone-density-related outcomes have also been examined in some AIS exercise studies (24).

Mechanistically, exercise-induced loading may stimulate osteogenic responses through mechanotransduction-related pathways, including STAT3 signaling, integrin-mediated mechanotransduction, and osteocyte calcium signaling (33–35) Systemic endocrine and muscle-derived factors, including ghrelin-related pathways and myokines, may also contribute to bone metabolic regulation (36–39) However, bone metabolism was not a primary outcome of the present network meta-analysis, and the included Cobb angle evidence does not directly establish whether changes in bone mineral density or bone metabolism mediate spinal curvature changes in AIS. Therefore, these mechanisms should be interpreted as hypotheses rather than findings directly supported by the Cobb angle evidence synthesized in this review.

## 6 Conclusion

This systematic review and network meta-analysis suggest that non-specific exercise therapies may be associated with reductions in Cobb angle in adolescents with idiopathic scoliosis. In the primary network analysis, spinal flexibility training and core muscle group training appeared to have higher ranking probabilities than the other intervention categories. However, these findings should be interpreted cautiously because of substantial heterogeneity, small sample sizes, sparse network structure, concerns regarding transitivity, limited confidence in network evidence, variability in intervention classification, and the inclusion of non-randomized controlled studies.

The current evidence does not support definitive conclusions regarding the superiority of one non-specific exercise modality over another. Non-specific exercise programs may be considered accessible supportive conservative strategies for adolescents with mild-to-moderate AIS, particularly in school, community, or home-based settings. Future studies should prioritize adequately powered randomized controlled trials with standardized intervention protocols, clear reporting of exercise dose and curve severity, appropriate comparator groups, and long-term follow-up. Outcomes beyond Cobb angle, including trunk rotation, curve progression, function, quality of life, adherence, and adverse events, should also be reported to better determine the clinical value of non-specific exercise therapy in AIS management.

## Data Availability

The original contributions presented in the study are included in the article/Supplementary Material, further inquiries can be directed to the corresponding author.
