# Supplementary material for: Comparative effects of non-specific exercise therapies on adolescent idiopathic scoliosis: a systematic review and network meta-analysis
Source: Front Sports Act Living. 2026 Sep 25;8:1901060. doi: 10.3389/fspor.2026.1901060 (PMC13650006; doi:10.3389/fspor.2026.1901060)
Supplement: Supplementary file 1 [file Datasheet1.pdf]

Comparative Effects of Non-Specific Exercise Therapies on Adolescent Idiopathic Scoliosis: A Systematic Review and Network Meta-Analysis

Contents

1. Supplementary Table S1. The Detailed Search Strategy 2
2. Supplementary Table S2. Studies excluded after re-assessment and reasons for exclusion .. 4
3. Supplementary Table S3. Intervention eligibility adjudication and node classification rationale 5
4. Supplementary Table S4. Data audit table for Cobb angle quantitative synthesis 10
5. Supplementary Figure S1. Strict RCT-only sensitivity analysis of network meta-analysis results for Cobb angle 12

1. **Supplementary Table S1. The Detailed Search Strategy**

	EMBASE	Total
#1	('adolescent'/exp OR 'child'/exp OR adolescent:ti,ab OR teenager*:ti,ab OR youth:ti,ab)	4,851,478
#2	('scoliosis'/exp OR scoliosis:ti,ab OR "adolescent idiopathic scoliosis":ti,ab OR AIS:ti,ab)	85,519
#3	(('exercise therapy'/exp OR 'exercise'/exp OR 'physical therapy'/exp OR 'yoga'/exp OR 'pilates'/exp OR 'exercise movement techniques'/exp OR "aerobic exercise":ti,ab OR "core stability":ti,ab OR "spinal flexibility exercise":ti,ab OR pilates:ti,ab OR yoga:ti,ab OR "corrective exercise":ti,ab OR "therapeutic exercise":ti,ab) NOT (Schroth:ti,ab OR "Schroth method":ti,ab))	721,610
#4	#1 AND #2 AND #3	1,831

	PubMed	Total
#1	"Adolescent"[MeSH] OR "Child"[MeSH] OR adolescent[tiab] OR teenagers[tiab] OR youth[tiab]	3,644,877
#2	"Scoliosis"[MeSH] OR scoliosis[tiab] OR "adolescent idiopathic scoliosis"[tiab] OR AIS[tiab]	53,047
#3	(("Exercise Therapy"[MeSH] OR "Exercise"[MeSH] OR "Physical Therapy Modalities"[MeSH] OR "Yoga"[MeSH] OR "Exercise Movement Techniques"[MeSH] OR aerobic exercise[tiab] OR core stability[tiab] OR spinal flexibility exercise[tiab] OR pilates[tiab] OR yoga[tiab] OR corrective exercise[tiab] OR therapeutic exercise[tiab]) NOT (Schroth[tiab] OR "Schroth Method"[tiab]))	445,391
#4	#1 AND #2 AND #3	835

	Web of Science	Total
#1	TS=(adolescent OR adolescents OR teenagers OR youth OR young people)	884,747
#2	TS=("adolescent idiopathic scoliosis" OR "idiopathic scoliosis" OR scoliosis OR "spinal deformity")	30,955
#3	TS=("exercise therapy" OR "physical exercise" OR "aerobic exercise" OR "core stability training" OR "core muscle training" OR "core strength training" OR yoga OR pilates OR "corrective exercise" OR "postural training") NOT TS=(Schroth)	74,211
#4	#1 AND #2 AND #3	73

	Cochrane Library	Total
#1	MeSH descriptor: [Scoliosis] explode all trees	819
#2	(scoliosis OR "spinal deformity" OR "adolescent idiopathic scoliosis" OR "idiopathic scoliosis"):ti,ab,kw	6,940
#3	MeSH descriptor: [Exercise Therapy] explode all trees	23,485

#4	(exercise OR "exercise therapy" OR "physical exercise" OR "aerobic exercise" OR "core stability training" OR "core muscle training" OR "core strength training" OR yoga OR pilates OR "corrective exercise" OR "postural training"):ti,ab,kw	153,478
#5	(Schroth):ti,ab,kw	142
#6	(adolescent OR adolescents OR teenager* OR youth OR "young people"):ti,ab,kw	180769
#7	(#1 OR #2) AND (#3 OR #4) AND #6 NOT #5	128

	**CNKI**	**Total**
#1	青少年 + 少年 + 青春期	330,976
#2	脊柱侧弯 + 脊柱侧凸 +' 青少年特发性脊柱侧弯 ' + AIS + 脊柱畸形	28,347
#3	(运动疗法 + 运动训练 + 运动干预 + 有氧运动 + 核心稳定性训练 + 核心肌群训练 + 核心力量训练 + 脊柱灵活性运动 + 瑜伽 + 普拉提 + 矫形体操) - (Schroth + 施罗斯)	97,618
#4	#1 * #2 * #3	195

	**WANFANG DATA**	**Total**
1#	(主题:(青少年 OR 少年 OR 青春期) and 主题:(脊柱侧弯 OR 脊柱侧凸 OR 青少年特发性脊柱侧弯 OR AIS OR 脊柱畸形) and 主题:(运动疗法 OR 运动训练 OR 运动干预 OR 有氧运动 OR 核心稳定性训练 OR 核心肌群训练 OR 核心力量训练 OR 脊柱灵活性运动 OR 瑜伽 OR 普拉提 OR 矫形体操)) not 主题:(Schroth OR 施罗斯))	231

	**Cqvip**	**Total**
1#	((((((((((((((题名或关键词=青少年 OR 题名或关键词=少年) OR 题名或关键词=青春期) AND ((((题名或关键词=脊柱侧弯 OR 题名或关键词=脊柱侧凸) OR 题名或关键词=青少年特发性脊柱侧弯) OR 题名或关键词=AIS) OR 题名或关键词=脊柱畸形))))) AND ((((((((((题名或关键词=运动疗法 OR 题名或关键词=运动训练) OR 题名或关键词=运动干预) OR 题名或关键词=有氧运动) OR 题名或关键词=核心稳定性训练) OR 题名或关键词=核心肌群训练) OR 题名或关键词=核心力量训练) OR 题名或关键词=脊柱灵活性运动) OR 题名或关键词=瑜伽) OR 题名或关键词=普拉提) OR 题名或关键词=矫形体操))))) AND (NOT (题名或关键词=Schroth OR 题名或关键词=施罗斯))))))	80

2. **Supplementary Table S2. Studies excluded after re-assessment and reasons for exclusion**

Study	Reason for exclusion after re-assessment	Excluded from systematic review	Excluded from pairwise meta-analysis	Excluded from network meta-analysis
Byung-Ki Lee et al., 2020	Participants were outside the predefined age range of 10–19 years; the reported participant age was 21 years.	Yes	Yes	Yes
Hanxue Cao et al., 2023	Participants were outside the predefined age range of 10–19 years; the reported participant age was 7–10 years.	Yes	Yes	Yes
Gozde Yagci et al., 2019	The experimental intervention combined core stabilization exercises with brace treatment, and the independent effect of the exercise component could not be isolated.	Yes	Yes	Yes
Wanchen Zhao et al., 2019	Participants were university students/young adults and were outside, or not clearly within, the predefined AIS age range of 10–19 years.	Yes	Yes	Yes
Kocaman H et al., 2021	The intervention consisted of core stabilization exercises combined with traditional training; therefore, the independent effect of the predefined non-specific exercise node could not be clearly isolated.	Yes	Yes	Yes

Note: After re-assessment according to the revised eligibility criteria, studies were excluded if participants were outside the predefined age range of 10–19 years, if the intervention included brace treatment or other active co-interventions whose independent exercise effect could not be isolated, or if the intervention could not be clearly classified as an eligible non-specific exercise therapy.

3. **Supplementary Table S3. Intervention eligibility adjudication and node classification rationale**

S3A：Eligibility adjudication

Author (Year)	Original intervention name	Key intervention components	Meets operational definition of non-specific exercise	Curve-specific auto-correction reported	Curve-type individualized prescription reported	Independent exercise effect isolatable	Sensitivity-analysis handling	Final eligibility and analysis decision
DENG L D（2021）	Core Muscle Group Training	Wall stretches, supine flexion–extension, equipment-assisted training	Yes	No formal curve-specific auto-correction reported	No	Yes	Retained in eligibility/classification sensitivity analysis.	Included in systematic review and primary Cobb-angle NMA.
Kwang-Jun（2017）	Core Muscle Group Training	Posterior pelvic tilt, curl-up, quadruped alternating lifts, forward-reaching walk	Yes	No formal curve-specific auto-correction reported	No	Yes, with minor reporting concern	Retained in eligibility/classification sensitivity analysis.	Included in systematic review and primary Cobb-angle NMA.
WANG K L（2024）	Core Muscle Group Training	Double bridge, single bridge, reverse bridge, equipment-assisted exercises	Yes	No formal curve-specific auto-correction reported	No	Yes	Retained in eligibility/classification sensitivity analysis.	Included in systematic review and primary Cobb-angle NMA.
LIU Z C（2024）	Spinal Stability Training	Plank, dynamic repetitions, unstable surface training, equipment-assisted	Yes	No formal curve-specific auto-correction reported	No	Yes	Excluded in the eligibility/classification sensitivity analysis because of moderate classification certainty	Included in systematic review and primary Cobb-angle NMA; examined in classification/eligibility sensitivity analysis because of moderate classification certainty.
ZENG F L（2020）	Core Muscle Group Training	Static holds, abdominal bridge, quadruped support, trunk rotation, crawling exercises	Yes	No formal curve-specific auto-correction reported	No	Yes, with minor reporting concern	Retained in eligibility/classification sensitivity analysis.	Included in systematic review and primary Cobb-angle NMA.
Monticone（2014）	Proprioceptive and Task-Oriented Training	Active postural correction, task-oriented practice, turning exercises	Borderline eligible	Active postural self-correction was reported; however, formal three-dimensional curve-specific auto-correction, such as Schroth-based rotational angular breathing, was not clearly reported.	Not clearly reported	Yes	Excluded in the eligibility/classification sensitivity analysis because of borderline eligibility.	Included in the systematic review and primary Cobb-angle NMA as a borderline eligible study because no formal three-dimensional curve-specific exercise protocol was clearly reported and the exercise effect was independently estimable. However, this study was excluded in the eligibility/classification sensitivity analysis because of borderline eligibility.
NIE D N（2025）	PNF / SPS / Combined	PNF combined with spiral muscle chain training to enhance	Unclear / borderline	Unclear	Not fully reported	Unclear; PNF and SPS	Not applicable; qualitative synthesis only.	Retained in qualitative synthesis only; not included in Cobb-angle

	Training	coordination and balance				components could not be clearly separated		quantitative synthesis or primary NMA because the independent non-specific exercise effect of the combined PNF/SPS programme could not be clearly separated.
LIU XH（2021）	Yoga Training	Stretching, core activation, meditation, and relaxation	Yes	No formal curve-specific auto-correction reported	No	Yes	Excluded in the eligibility/classification sensitivity analysis because of moderate classification certainty.	Included in systematic review and primary Cobb-angle NMA; examined in classification/eligibility sensitivity analysis because of moderate classification certainty.
Fishman（2021）	Yoga-based isometric exercise	Side plank, half-moon pose, elevated side plank	Borderline eligible	No formal three-dimensional curve-specific auto-correction was reported; however, curve-related posture selection could not be fully excluded.	Unclear / possibly related to curve characteristics	Yes, with methodological concerns	Excluded in the eligibility/classification sensitivity analysis because of borderline eligibility and low classification certainty.	Included in the systematic review and primary Cobb-angle NMA as a borderline eligible study because no formal three-dimensional curve-specific exercise protocol was clearly reported and the exercise effect was independently estimable. However, this study was excluded in the eligibility/classification sensitivity analysis because of borderline eligibility.
Manzak（2024）	Pilates Training	Basic Pilates training (hybrid remote and home-based)	Yes	No formal curve-specific auto-correction reported	No	Yes	Retained in eligibility/classification sensitivity analysis.	Included in systematic review and primary Cobb-angle NMA.
LIU L（2023）	General corrective exercise	Breathing, flexibility, and core combination exercises	Yes	No formal curve-specific auto-correction reported	No	Yes	Excluded in the eligibility/classification sensitivity analysis because of moderate classification certainty.	Included in the systematic review and primary Cobb-angle NMA because no formal curve-specific auto-correction or curve-type individualized prescription was reported. The study was examined in the eligibility/classification sensitivity analysis because of moderate classification certainty.
FU T（2018）	General corrective exercise	Stretching and strengthening combination exercises	Borderline eligible, qualitative only	No formal curve-specific auto-correction reported	No	Yes	Not applicable; qualitative synthesis only.	Retained in qualitative synthesis only; not included in Cobb-angle quantitative synthesis or primary NMA.

Xavier（2020）	Aerobic and Resistance Training	Running, cycling, and upper/lower limb resistance training	Yes	No formal curve-specific auto-correction reported	No	Yes	Not applicable; qualitative synthesis only.	Retained in qualitative synthesis only; not included in Cobb-angle quantitative synthesis or primary NMA.
Lau（2021）	HIIT（E-Fit）	Home-based high-intensity interval training	Yes	No formal curve-specific auto-correction reported	No	Yes, with bracing-related concern	Not applicable; qualitative synthesis only.	Retained in qualitative synthesis only; not included in Cobb-angle quantitative synthesis or primary NMA.

S3B. Network-node classification and sensitivity-analysis handling

Author (Year)	Original intervention name	Key intervention components	Assigned network node	Basis for classification	Classification certainty	Sensitivity-analysis handling
DENG L D（2021）	Core Muscle Group Training	Wall stretches, supine flexion–extension, equipment-assisted training	Core Muscle Group Training	The intervention mainly involved trunk/core strengthening and stabilization exercises. No formal curve-specific three-dimensional scoliosis correction was reported.	High	Retained in eligibility/classification sensitivity analysis.
Kwang-Jun（2017）	Core Muscle Group Training	Posterior pelvic tilt, curl-up, quadruped alternating lifts, forward-reaching walk	Core Muscle Group Training	The intervention mainly targeted core stabilization and trunk muscle control through pelvic tilt, curl-up, and quadruped exercises.	High	Retained in eligibility/classification sensitivity analysis.
WANG K L（2024）	Core Muscle Group Training	Double bridge, single bridge, reverse bridge, equipment-assisted exercises	Core Muscle Group Training	The intervention mainly consisted of bridge-based and equipment-assisted trunk stabilization exercises.	High	Retained in eligibility/classification sensitivity analysis.
LIU Z C（2024）	Spinal Stability Training	Plank, dynamic repetitions, unstable surface training, equipment-assisted	Core Muscle Group Training	Although described as spinal stability training, the reported components mainly involved plank, dynamic core exercises, unstable surface training, and trunk stabilization.	Moderate	Excluded in the eligibility/classification sensitivity analysis because of moderate classification certainty
ZENG F L（2020）	Core Muscle Group Training	Static holds, abdominal bridge, quadruped support, trunk rotation, crawling exercises	Core Muscle Group Training	The intervention mainly targeted core strength and trunk stability through static holds, abdominal bridge, quadruped support, trunk rotation, and crawling exercises.	High	Retained in eligibility/classification sensitivity analysis.
Monticone（2014）	Proprioceptive and Task-Oriented Training	Active postural correction, task-oriented practice, turning exercises	Proprioceptive Training	The intervention emphasized postural control, proprioception, and task-oriented motor training. However, active postural correction may overlap with scoliosis-specific approaches, creating classification uncertainty.	Moderate	Excluded in the eligibility/classification sensitivity analysis because of borderline eligibility.
NIE D N（2025）	PNF / SPS / Combined Training	PNF combined with spiral muscle chain training to enhance coordination and	Proprioceptive Training	The intervention mainly targeted proprioception, coordination, balance, and motor control	Low	Not applicable; qualitative synthesis only.

		balance		through PNF and spiral stabilization training. However, the combined SPS component may overlap with scoliosis-specific or structured corrective approaches.		
LIU XH（2021）	Yoga Training	Stretching, core activation, meditation, and relaxation	Spinal Flexibility Training	The intervention mainly involved general yoga-based stretching, core activation, meditation, and relaxation, without clearly reported formal curve-specific scoliosis correction.	Moderate	Excluded in the eligibility/classification sensitivity analysis because of moderate classification certainty.
Fishman（2021）	Yoga-based isometric exercise	Side plank, half-moon pose, elevated side plank	Spinal Flexibility Training	The intervention used yoga-based isometric postures and was not reported as Schroth-based. However, posture selection may have been related to curve characteristics, so classification uncertainty remained.	Low	Excluded in the eligibility/classification sensitivity analysis because of borderline eligibility and low classification certainty.
Manzak（2024）	Pilates Training	Basic Pilates training (hybrid remote and home-based)	Spinal Flexibility Training	The intervention involved general Pilates-based flexibility, mobility, and trunk-control exercises delivered through hybrid telerehabilitation, without reported formal curve-specific correction.	High	Retained in eligibility/classification sensitivity analysis.
LIU L（2023）	General corrective exercise	Breathing, flexibility, and core combination exercises	General Corrective Exercise	The intervention combined general breathing, flexibility, and core exercises. Because these components may overlap with corrective exercise principles, classification uncertainty remained.	Moderate	Excluded in the eligibility/classification sensitivity analysis because of moderate classification certainty.
FU T（2018）	General corrective exercise	Stretching and strengthening combination exercises	General Corrective Exercise	The intervention consisted of general stretching and strengthening gymnastics. It was not clearly reported as a formal scoliosis-specific protocol, but the term “corrective exercise” creates some classification uncertainty.	Low	Not applicable; qualitative synthesis only.
Xavier（2020）	Aerobic and Resistance Training	Running, cycling, and upper/lower limb resistance training	Aerobic Exercise	The intervention consisted of general aerobic and resistance training, including running, cycling, and limb resistance exercises, without curve-specific prescription.	High	Not applicable; qualitative synthesis only.
Lau（2021）	HIIT（E-Fit）	Home-based high-intensity interval training	Aerobic Exercise	The intervention was a general home-based high-intensity interval training program and was not individualized according to curve type.	High	Not applicable; qualitative synthesis only.

Note: PNF, proprioceptive neuromuscular facilitation; SPS, spiral stabilization; HIIT,

high-intensity interval training. Classification certainty was judged according to whether the intervention content clearly matched the assigned network node, whether formal curve-specific three-dimensional scoliosis correction was reported, and whether the intervention could be clearly distinguished from scoliosis-specific exercise. Interventions with moderate or low classification certainty were considered in the eligibility/classification sensitivity analysis. Some studies were included in the qualitative synthesis only and did not contribute to the Cobb angle network meta-analysis.

**S3C. Summary of eligibility/classification sensitivity analysis**	
Item	Result
Sensitivity-analysis rule	Studies with borderline eligibility or moderate-to-low classification certainty were excluded.
Studies excluded from the sensitivity-analysis dataset	Liu ZC 2024; Monticone 2014;<br>Liu XH 2021; Fishman 2021; Liu L 2023.
Studies retained	Deng LD 2021; Kwang-Jun 2017;<br>Wang KL 2024; Zeng FL 2020; Manzak 2024.
Nodes retained	Routine Care/Exercise; Core Muscle Group Training; Spinal Flexibility Training.
Nodes no longer represented	Proprioceptive Training; General Corrective Exercise.
Interpretation	After exclusion of borderline or classification-uncertain studies, the network became sparse and complete ranking across all original intervention nodes was no longer reliable.

4. Supplementary Table S4. Data audit table for Cobb angle quantitative synthesis

Supplementary Table S4. Data audit table for Cobb angle quantitative synthesis

Study	Study design	Intervention node	Experimental n	Control n	Total n	Pairwise meta-analysis	Network meta-analysis	RCT-only sensitivity analysis	Reason if excluded
DENG L D（2021）	Non-randomized controlled study	Core Muscle Group Training	49	49	98	Yes	Yes	No	Excluded from RCT-only sensitivity analysis because it was non-randomized.
Kwang-Jun（2017）	Non-randomized controlled study	Core Muscle Group Training	14	15	29	Yes	Yes	No	Excluded from RCT-only sensitivity analysis because it was non-randomized.
WANG K L（2024）	Non-randomized controlled study	Core Muscle Group Training	18	15	33	Yes	Yes	No	Excluded from RCT-only sensitivity analysis because it was non-randomized.
LIU Z C（2024）	RCT	Core Muscle Group Training	20	20	40	Yes	Yes	Yes	Not applicable.
ZENG F L（2020）	RCT	Core Muscle Group Training	40	40	80	Yes	Yes	Yes	Not applicable.
Monticone（2014）	RCT	Proprioceptive Training	55	55	110	Yes	Yes	Yes	Not applicable.
NIE D N（2025）	RCT	Proprioceptive Training	24	8	32	No	No	No	Retained in qualitative synthesis only; data were not compatible with the final Cobb angle quantitative synthesis.
LIU XH（2021）	RCT	Spinal Flexibility Training	8	8	16	Yes	Yes	Yes	Not applicable.
Fishman（2021）	Non-randomized controlled study	Spinal Flexibility Training	41	15	56	Yes	Yes	No	Excluded from RCT-only sensitivity analysis because it was non-randomized.
Manzak（2024）	RCT	Spinal Flexibility Training	16	15	31	Yes	Yes	Yes	Not applicable.
Xavier（2020）	RCT	Aerobic Exercise	20	20	40	No	No	No	Retained in qualitative synthesis only; Cobb angle data were not included in the final quantitative synthesis.
Lau（2021）	RCT	Aerobic Exercise	20	20	40	No	No	No	Retained in qualitative synthesis only; Cobb angle was not available as an extractable outcome for quantitative synthesis.
LIU L（2023）	Non-randomized controlled study	General Corrective Exercise	48	48	96	Yes	Yes	No	Excluded from RCT-only sensitivity analysis because it was non-randomized.
FU T（2018）	Non-randomized controlled study	General Corrective Exercise	11	11	22	No	No	No	Retained in qualitative synthesis only; extractable Cobb angle data suitable for quantitative synthesis were unavailable or incompatible with the final analysis.

Note: RCT, randomized controlled trial. The Cobb angle pairwise meta-analysis and primary

network meta-analysis were based on the same final dataset of 10 studies and 589 participants. Studies included in the systematic review but not in the Cobb angle quantitative synthesis were retained for qualitative synthesis only because extractable post-intervention Cobb angle data were unavailable or incompatible with the final analysis. The strict RCT-only sensitivity analysis excluded all non-randomized controlled studies; therefore, the general corrective exercise node disappeared.

Summary of analytic datasets

Analysis	Number of studies	Experimental n	Control n	Total n	Notes
Systematic review	14	384	339	723	All eligible studies included in qualitative synthesis.
Cobb angle pairwise meta-analysis	10	309	280	589	Included studies with extractable post-intervention Cobb angle data.
Cobb angle network meta-analysis	10	309	280	589	Based on the same final dataset as the pairwise meta-analysis.
Strict RCT-only sensitivity analysis	5	139	138	277	Non-randomized studies were excluded; the general corrective exercise node disappeared.

5. Supplementary Figure S1. Strict RCT-only sensitivity analysis of network meta-analysis results for Cobb angle

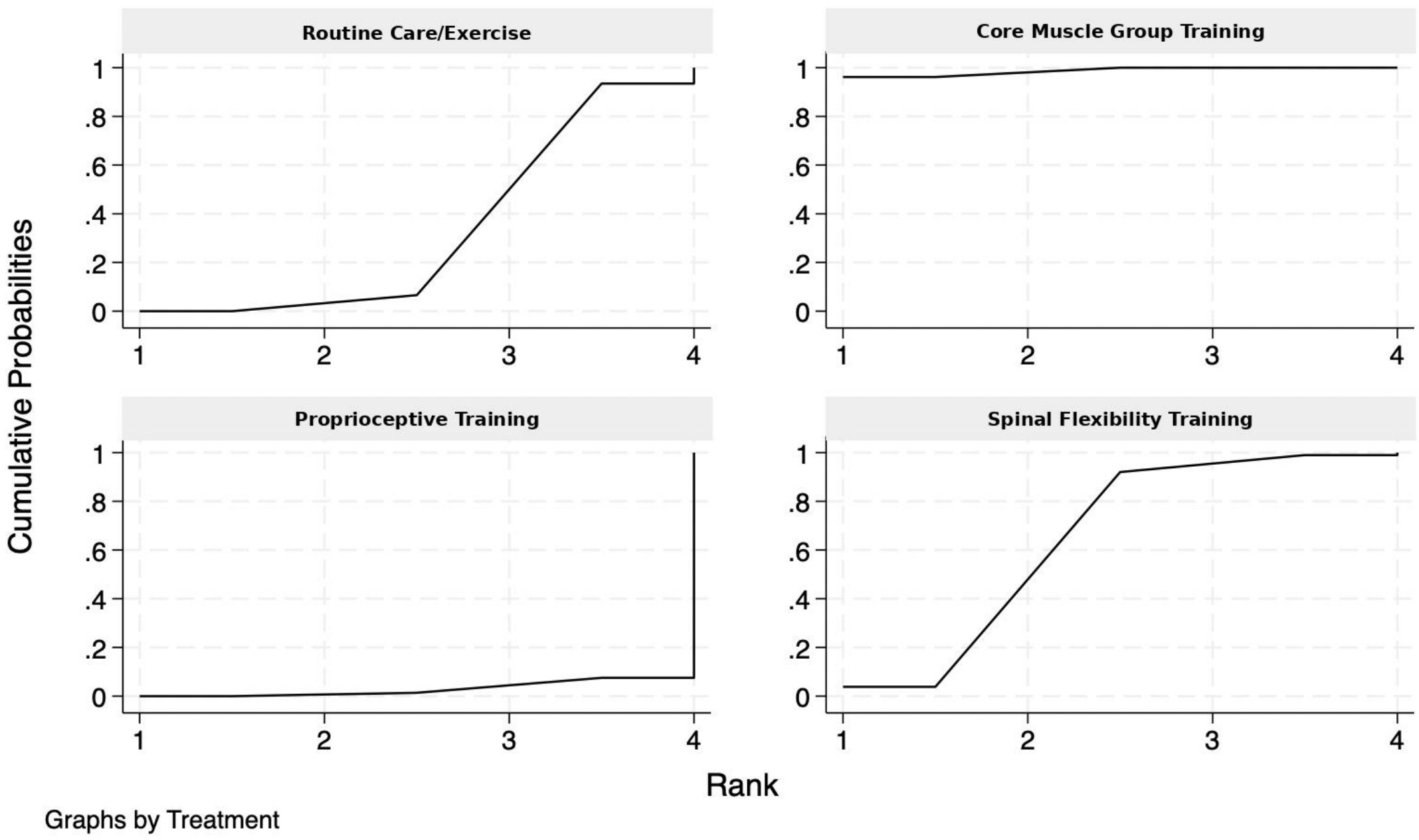

Treatm~t	SUCRA	PrBest	MeanRank
Routine Care/ Exercise	33.4	0.0	3.0
Core Muscle Group Training	98.7	96.2	1.0
Proprioceptive Training	3.0	0.0	3.9
Spinal Flexibility Training	65.0	3.8	2.1

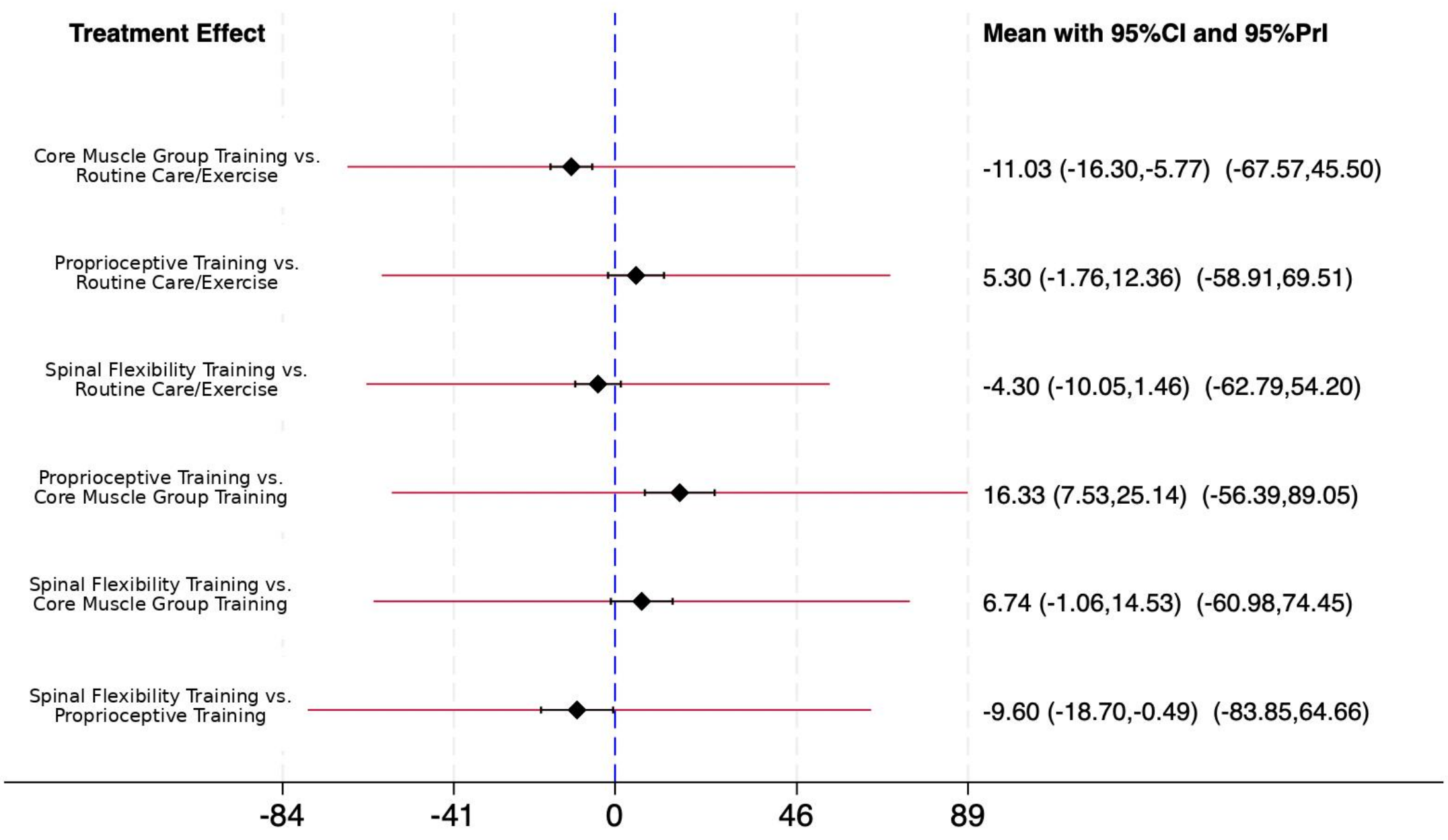

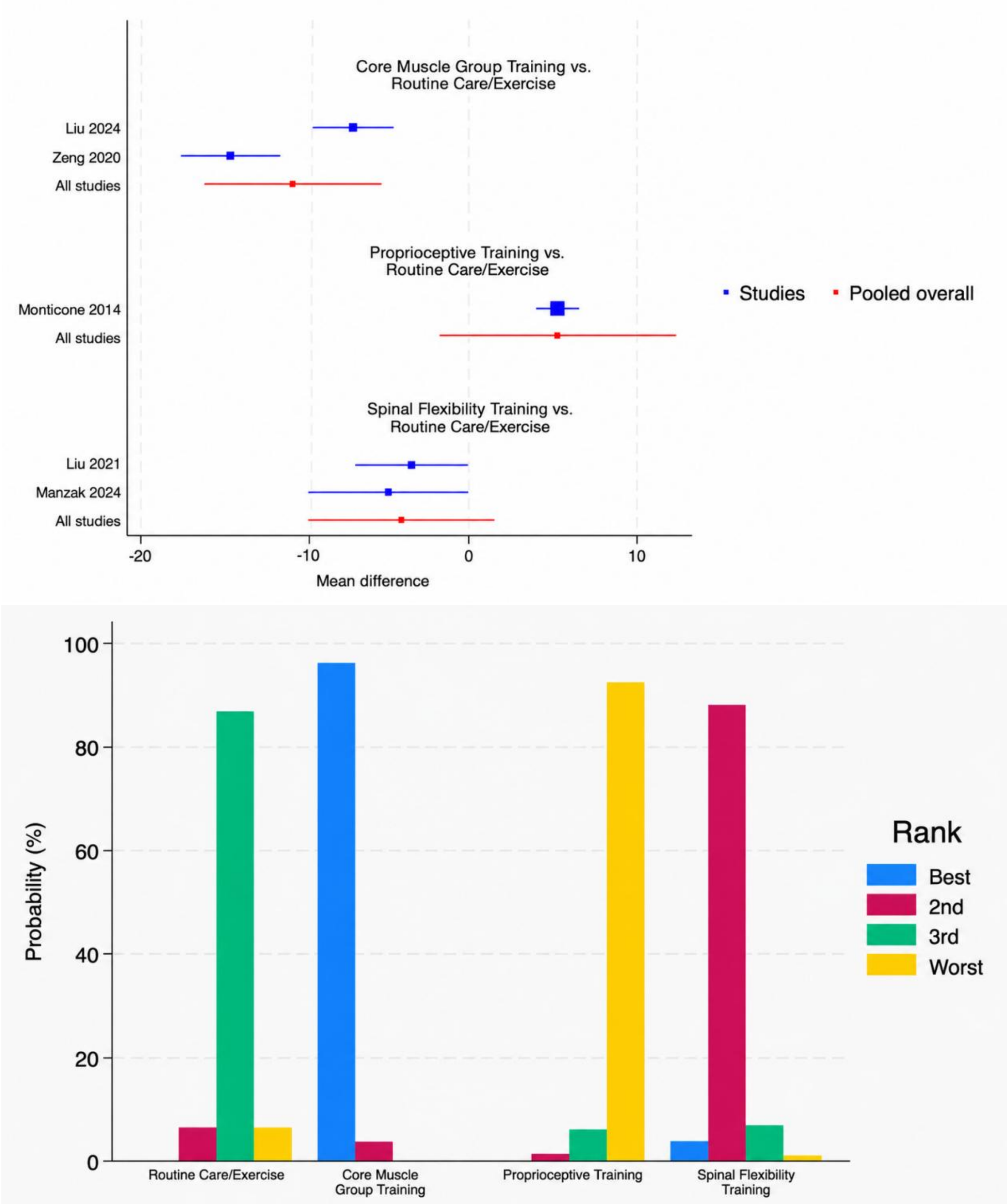

Supplementary Figure S1. Strict RCT-only sensitivity analysis of network meta-analysis results for Cobb angle. Non-randomized controlled studies were excluded from this analysis. After exclusion, the general corrective exercise node disappeared, and the RCT-only network included routine care/exercise, core muscle group training, proprioceptive training, and spinal flexibility training. CI, confidence interval; MD, mean difference; PrI, prediction interval; SUCRA, surface under the cumulative ranking curve.
